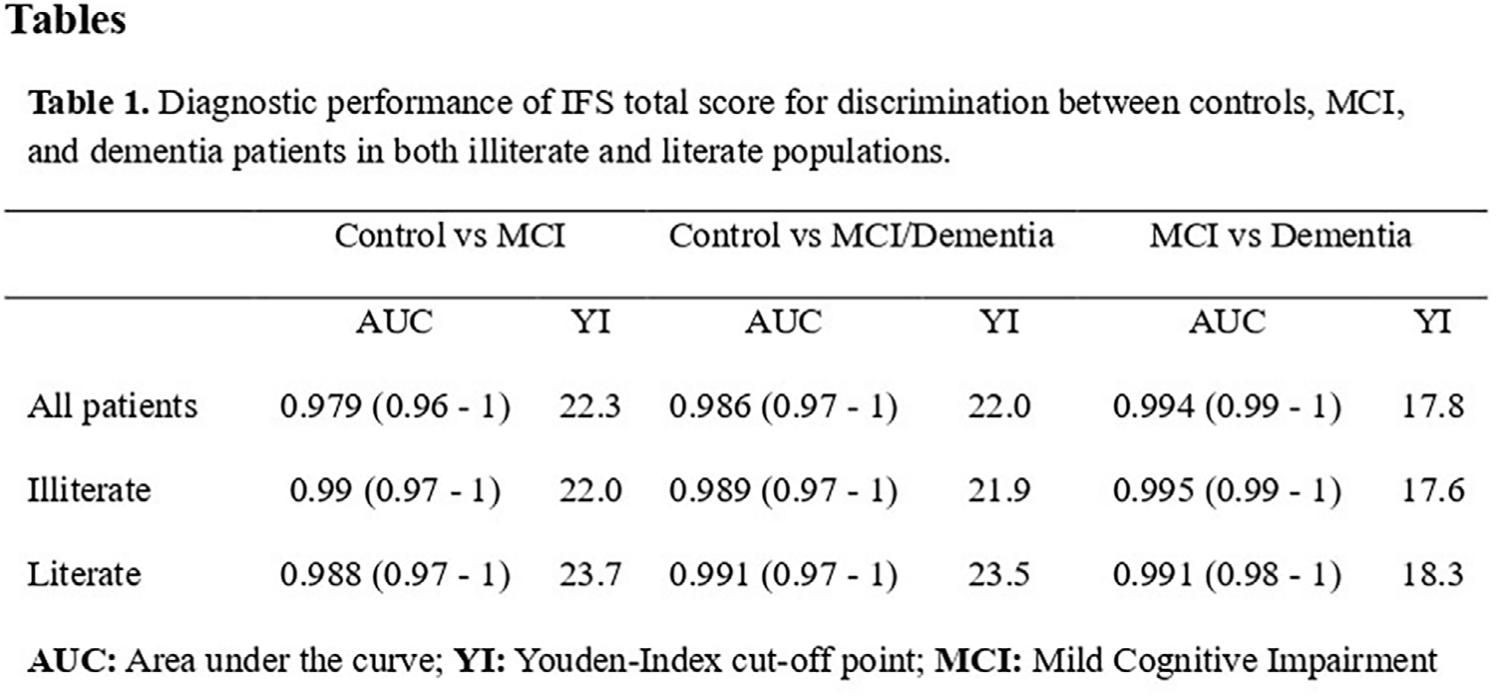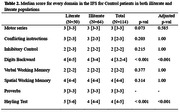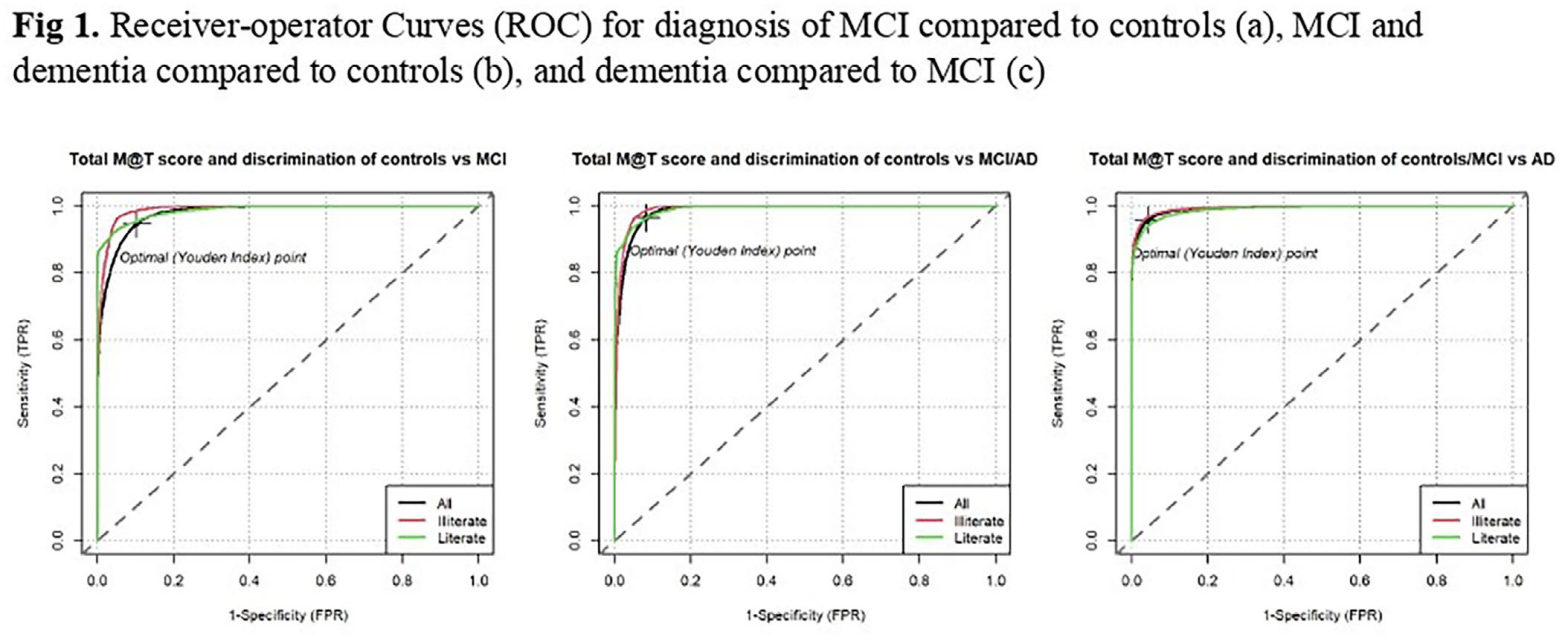# Comparing the diagnostic performance of the INECO Frontal Screening in illiterate vs. literate older adults with cognitive impairment in Lima, Peru

**DOI:** 10.1002/alz.092000

**Published:** 2025-01-03

**Authors:** Nilton Custodio, Marco Malaga, Rosa Montesinos, Arturo Jhonny Ruiz‐Yaringaño, Fiorella Baca, Diego Chambergo‐Michilot, Graciet Verástegui, Katherine Agüero‐Flores, Rossana Cruzdel Castillo, José Cuenca, José Carlos Huilca, Serggio Lanata, Diego Bustamante‐Paytan

**Affiliations:** ^1^ Cognitive Impairment Diagnosis and Dementia Prevention Unit, Peruvian Institute of Neurosciences, Lima, Lima Peru; ^2^ Cognitive Impairment Diagnosis and Dementia Prevention Unit, Instituto Peruano de Neurociencias, Lima, Perú, Lima, Lima Peru; ^3^ Sociedad Científica de San Fernando, Lima, Lima Peru; ^4^ Cognitive Impairment and Dementia Prevention Unit, Peruvian Institute of Neurosciences, Lima, Lima Peru; ^5^ Unit Cognitive Impairment and Dementia Prevention, Peruvian Institute of Neurosciences, Lima, Peru, Lima, Lima Peru; ^6^ Cognitive impairment diagnosis and dementia prevention unit, Instituto Peruano de Neurociencias, Lima Peru; ^7^ Memory and Aging Center, UCSF Weill Institute for Neurosciences, University of California, San Francisco, San Francisco, CA USA

## Abstract

**Background:**

Rising dementia prevalence in Low and Middle‐Income Countries (LMICs), fueled by socio‐economic disparities, necessitates accessible brief cognitive tests. The INECO Frontal Screening (IFS) proves effective for detection of Mild Cognitive Impairment (MCI) and early dementia. It prioritizes executive functions, is suitable for low‐educated or illiterate individuals, and robust diagnostic capacity.

**Method:**

We conducted a secondary analysis of a prior patient cohort. Illiterate participants met the following criteria: proficiency in Spanish for over 10 years, age ≥ 60, less than 1 year of formal education, and inability to read and write. Literate patients were aged 60 or older, Spanish speakers, and had a minimum of 6 years of formal education. A clinical evaluation with three consecutive phases was used as gold standard diagnosis, resulting in a final classification based on international criteria. The IFS was applied to all participants. To compare the diagnostic performance of the IFS, a Receiver‐Operator Curve (ROC) analysis was conducted. The average scores for each IFS domain were compared among all groups in each cohort using a Wilcoxon test.

**Result:**

We included 114 controls, 101 MCI patients, and 105 all type dementia patients. Median age was 68 (67‐71), 69 (66‐71) and 72 (71‐75), respectively, and female participants accounted for 45.6%, 47.5% and 45.7% for each group, respectively. An excellent diagnostic performance was found when we evaluated the IFS Total score for discrimination between controls, MCI, and dementia patients across both groups (Fig. 1). The analysis based on the Youden index showed that, the optimal cut‐off points for MCI (22 vs 23.7), and dementia patients (17.6 vs 18.3), was lower in illiteracy participants (Table 1). Finally, when evaluating each IFS domain among control patients in both groups, statistically significant differences were found in Digits backward and the Hayling test (p‐value <0.001) (Table 2).

**Conclusion:**

The IFS seems to be useful in assessing both illiterate and literate older adult patients for the presence of MCI and dementia in LMIC settings.